# Standard (8 weeks) vs long (12 weeks) timing to minimally-invasive surgery after NeoAdjuvant Chemoradiotherapy for rectal cancer: a multicenter randomized controlled parallel group trial (TiMiSNAR)

**DOI:** 10.1186/s12885-019-6271-3

**Published:** 2019-12-16

**Authors:** Igor Monsellato, Filippo Alongi, Elisa Bertocchi, Stefania Gori, Giacomo Ruffo, Elisa Cassinotti, Ludovica Baldarti, Luigi Boni, Graziano Pernazza, Fabio Pulighe, Carlo De Nisco, Roberto Perinotti, Emilio Morpurgo, Tania Contardo, Enzo Mammano, Ugo Elmore, Roberto Delpini, Riccardo Rosati, Federico Perna, Andrea Coratti, Benedetta Menegatti, Sergio Gentilli, Paolo Baroffio, Piero Buccianti, Riccardo Balestri, Cristina Ceccarelli, Valter Torri, Davide Cavaliere, Leonardo Solaini, Giorgio Ercolani, Elena Traverso, Vittorio Fusco, Maura Rossi, Fabio Priora, G. Numico, Paola Franzone, Sara Orecchia

**Affiliations:** 1 Azienda Ospedaliera SS. Antonio e Biagio e Cesare Arrigo, Alessandria, Italy; 2 Ospedale Sacro Cuore Don Calabria, Negrar, Italy; 3 Department of Surgery, Fondazione IRCCS Ca’ Granda, Ospedale Maggiore Policlinico, University of Milan, Milan, Italy; 4 Azienda Ospedaliera San Giovanni Addolorata, Rome, Italy; 5 Ospedale San Francesco, Nuoro, Italy; 6 Ospedale degli Infermi, Biella, Italy; 7 Ospedale Civile Pietro Cosma, Camposampiero/Ospedale Sant’Antonio, Padova, Italy; 8 Ospedale Civile Pietro Cosma, Padova, Camposampiero Italy; 9 Ospedale San raffaele IRCCS, Milan, Italy; 10 Azienda Ospedaliero Universitaria Careggi, Florence, Italy; 11 Azienda Ospedaliero Universitaria Maggiore Della Carità, Novara, Italy; 12 Azienda Ospedaliero Universitaria Pisana, Pisa, Italy; 13 Istituto di Ricerche Farmacologiche Mario Negri IRCCS, Milan, Italy; 14 Ospedale G.B. Morgagni L. Pierantoni, Forlì, Italy

**Keywords:** Radiation therapy, Minimally invasive surgery, Rectal cancer, Neoadjuvant treatment, Robotic surgery, TaTME, Timing to surgery

## Abstract

**Background:**

The optimal timing of surgery in relation to chemoradiation is still controversial. Retrospective analysis has demonstrated in the recent decades that the regression of adenocarcinoma can be slow and not complete until after several months. More recently, increasing pathologic Complete Response rates have been demonstrated to be correlated with longer time interval. The purpose of the trial is to demonstrate if delayed timing of surgery after neoadjuvant chemoradiotherapy actually affects pathologic Complete Response and reflects on disease-free survival and overall survival rather than standard timing.

**Methods:**

The trial is a multicenter, prospective, randomized controlled, unblinded, parallel-group trial comparing standard and delayed surgery after neoadjuvant chemoradiotherapy for the curative treatment of rectal cancer. Three-hundred and forty patients will be randomized on an equal basis to either robotic-assisted/standard laparoscopic rectal cancer surgery after 8 weeks or robotic-assisted/standard laparoscopic rectal cancer surgery after 12 weeks.

**Discussion:**

To date, it is well-know that pathologic Complete Response is associated with excellent prognosis and an overall survival of 90%. In the Lyon trial the rate of pCR or near pathologic Complete Response increased from 10.3 to 26% and in retrospective studies the increase rate was about 23–30%. These results may be explained on the relationship between radiation therapy and tumor regression: DNA damage occurs during irradiation, but cellular lysis occurs within the next weeks. Study results, whether confirmed that performing surgery after 12 weeks from neoadjuvant treatment is advantageous from a technical and oncological point of view, may change the current pathway of the treatment in those patient suffering from rectal cancer.

**Trial registration:**

ClinicalTrials.gov NCT3465982.

## Background

Chemoradiotherapy is a well-known risk reducing treatment of local recurrence in the treatment of rectal cancer, followed by total mesorectal excision (TME). In low rectal tumors, surgery alone has the 30% overall survival and a local recurrence rate of about 55–65%, with a disease-free survival of 30–35% [1]. Preoperative administration of fluorouracil-based chemotherapy improved local recurrence rates to 7% [2]. The optimal timing of surgery in relation to chemoradiation is still controversial. Retrospective analysis has demonstrated in the recent decades that the regression of adenocarcinoma can be slow and not complete until after several months [3]. More recently, increasing pCR (pathological complete response) rates have been demonstrated to be correlated with longer time interval [4–6]. Conversely, several reports have shown no impact of the interval after chemoradiation on pCR and technical performance [7, 8]. In the Lyon trial the rate of pCR or near pCR increased from 10.3 to 26% [9] and in retrospective studies the increase rate was about 23–30%. These results may be explained on the relationship between radiation therapy and tumor regression: DNA (DeoxyriboNucleic Acid) damage occurs during irradiation, but cellular lysis occurs within the next weeks [10]. A recent pilot study on comparison of resonance imaging and histopathological responses at two times, has suggested that volume reduction and down-staging occur between week 9 and week 14 after neoadjuvant treatment, with a 23% pCR rate at longer time [11]. In the Stockholm III trial, a significantly lower frequency of postoperative complications was reported, even though not described in the other studies where morbidity and complications were the same. All of these studies, however, presented some biases, such as absence of randomization, the choice of surgical timing made arguably by the surgeon, tumor size and response to RCT (radiochemotherapy), different cut-off period and a limited number of recruited patients, that may have negatively or positively influenced these results [12, 13]. Delaying surgery with the aim to detect excellent responders for organ preservation, eventually, may be legitimate, even though the start of adjuvant therapy, whose advantage in pretreated rectal cancer patients is still controversial, would be delayed, and this may negatively affect survival [14, 15]. A recent meta-analysis on thirteen reports has been published, showing rates of 14 and 20% in the shorter and longer group, respectively. This meta-analysis has some biases: the pCR correlation with surgical delay could not be adjusted in a multivariate analysis with other clinico-pathological variables, the outcome (DFS and OS) of pCR, even if likely better than those without pCR as literature demonstrates, could not be directly assessed due to lack of individual patient data, the number of patients operated on in the delayed group could have been chosen using a surgical decision, different time intervals were grouped all together, no randomized trial were included in the meta-analysis, and the relevance of the reports included in was assessed by NOS scale (Newcastle–Ottawa scale), that is quite arbitrary, several reports on observation, demonstrating a higher percentage of pCR, were not included, but it is quite relevant to consider also these studies. TiMiSNAR has been developed to improve and define previous results from retrospective and review analyses.

## Methods/design

**Fig. 1:**
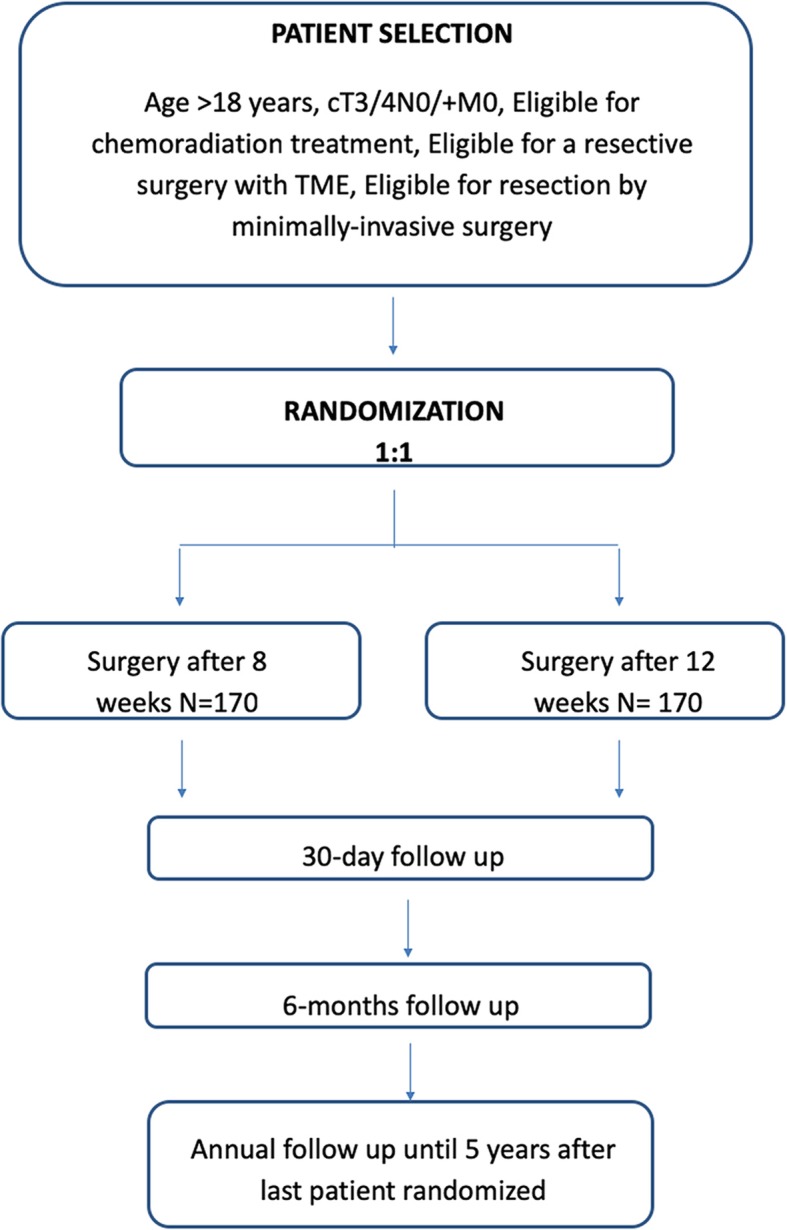
Flow chart of TiMiSNAR Trial

The trial is a multicenter, prospective, randomized controlled, unblinded, parallel-group trial comparing standard and delayed surgery after neoadjuvant chemoradiotherapy for the curative treatment of rectal cancer. Three-hundred and forty patients will be randomized on an equal basis to either robotic-assisted/standard laparoscopic rectal cancer surgery after 8 weeks or robotic-assisted/standard laparoscopic rectal cancer surgery after 12 weeks (Fig. 1). Eight weeks are the current standard interval to surgery after neodjuvant treatment, while 12 weeks represent the “minimum” longer time interval to determine further tumor modifications and the “a priori” choice to avoid hypothetic surgical detrimental effect (postoperative complications related to radiation therapy). The recruiting interval will be of 5 years and the follow-up period will end 5 years after the last patient is randomized.

The trial has been held in Alessandria at SS. Antonio e Biagio e Cesare Arrigo Community Hospital, Italy and in others National Academic and not-Academic Centers, list of which is available at https://www.timisnar.it.

The Primary Endpoint is pCR; secondary endpoints are: DFS (disease-free survival), OS (overall survival), postoperative complications (Clavien-Dindo classification), reintervention, late complications (Clavien-Dindo classification), radiation toxicity, chemotherapy toxicity, QoL (quality of life), Functional status.

Inclusion Criteria are: age > 18 years, cT3/4 N0/+M0 confirmed on CT-scan (Computed Tomography Scan), MRI (Magnetic Resonance Imaging - stratification for T3a-b-c-d), tumor starting from the distal or medium rectum (even those crossing the peritoneal reflection at distal margin, within 15 cm from the anal margin), histologically-proven adenocarcinoma of the rectum, eligible for a resective surgery with TME (low anterior resection, intersphyncteric resection, abdominoperineal resection), eligible for resection by minimally-invasive surgery (standard or robotic-assisted laparoscopic procedure, all robotic systems will be accepted), eligible for chemoradiation treatment, able to give written informed consent, capable of completing required questionnaires at time of consent (provided questionnaires are available in a language spoke fluently by the participant).

Main exclusion Criteria are: metastatic disease, squamous carcinoma of the anal canal, unable to complete neoadjuvant treatment.

Patients will be randomized on a 1:1 basis to receive minimally-invasive rectal cancer surgery 8 or 12 weeks after neoadjuvant treatment and will be allocated a unique trial number.

Participants will be randomized using Sealed Envelope Ltd. 2017 Online Simple randomization service. Allocation concealment will be ensured, as the service will not release the randomization code until the patient has been recruited into the trial, which takes place after all baseline measurements have been completed.

An informed consent to participate has been prepared and will be obtained by all the participants.

All patients who give consent for participation and who fulfil the inclusion criteria will be randomized. Randomization will be requested by the staff member responsible for recruitment and clinical interviews from all participating centers. Due to the nature of the intervention neither participants nor staff can be blinded to allocation, but are strongly inculcated not to disclose the allocation status of the participant at the follow up assessments.

All the involved centers have to respect the following criteria: site able to perform robotic-assisted and standard laparoscopic rectal cancer surgery and TaTME (transanal total mesorectal excision); site able to provide standard neoadjuvant treatment, both chemo and radiation therapy; predicted capability to recruit a minimum of 15 patients per year to the trial.

Neoadjuvant treatment consists in long course radiation therapy with IMRT (Intensity Modulated RadioTherapy – 50-54 Gy in 25–28 fractions; an optional boost is suggested) associated to concomitant chemotherapy treatment (Capecitabine 825 mg /m2/ twice daily during radiation therapy).

Several studies have compared IMRT of rectal cancer to 3D Conformal Radiotherapy. Although results from comparative randomized clinical trial are not available yet, IMRT is usually associated with less dose to organ at risk, such as urinary bladder, small bowel and anal sphincters (in selected cases). This is translated into better clinical outcomes, in terms of gastrointestinal toxicity, genitourinary toxicity and skin side effects [16–20].

### Restaging and treatment-efficacy assessment after Neoadjuvant therapy

The MERCURY study group has developed an MRI-based tumor regression grading (ymrTRG) system by applying the principles of histopathological tumor regression grade (ypTRG) [21].

Recently, a pilot study from UK has defined two groups of patients divided into favourable vs unfavourable responders based on the following three factors:

- ymrT
- ymrTRG
- Change in volume

ymrT is based on the interpretation of local extent of persistent tumor signal intensity relative to the layers of bowel wall on T2-weighted images. Tumor response is evaluated as either replacement of tumor signal by low signal intensity fibrosis (dark stroma) or the development of high signal intensity mucin pools, that are not considered to be tumor.

ymrTRG is based on principles similar to the pathological ypTRG system described by Dworak and subsequently modified by Mandard.

Change in volume, better defined as percentage volume reduction is calculated multiplying tumor length, width and height, using the following formula:

$$ 100\ast \left\{\left(\mathrm{Volume}\ \mathrm{at}\ \mathrm{baseline}\right)-\left(\mathrm{Volume}\ \mathrm{post}-\mathrm{CRT}\right)\right\}/\left(\mathrm{Volume}\ \mathrm{at}\ \mathrm{baseline}\right) $$

Time interval to surgery in this trial are 8 weeks and 12 weeks after treatment, that are the standard and the expected “minimum” longer time interval to determine further tumor modifications. Post-treatment staging for evaluation of postneoadjuvant treatment response, eventually, will depend on MRI evaluation at week 7 for patients in both the two arms; a MRI evaluation will be repeated at week 11 for patients randomized in the delayed arm.

A Thoraco-abdominal CT-Scan with and without contrast enhancement will be performed at week 6 after neoadjuvant surgery, for restaging of potential disseminated disease.

All MRI exams are collected and sent to the Promoting Center for final revision by a well-trained Pelvic MRI expert radiologist. Every participating center must fill in a structured MRI form according to the fac-simile provided by the ESGAR (European Society of Gastrointestinal and Abdominal Radiology) [22].

### Surgery

Minimally-invasive mesorectal resection is required: both robotic or standard laparoscopic approach or TaTME will be accepted, in accordance with each surgeon’s usual practice. The specifics of each operation will be at the discretion of the operating surgeon (e.g. port-site placement, mobilization of the splenic flexure, inferior mesenteric artery/vein division, high versus low vascular division etc.), as well as the decision to convert to an open operation. Conversion to open operation is defined as the use of a laparotomy wound for any part of the mesorectal dissection. All participating centers are allowed and suggested to use Indocyanine Green test (ICG), wherever available, but it is not mandatory. Several studies have shown that ICG test could reduce anastomotic leakage and thus postoperative complications, that are important in light of the secondary endpoints. A recent systematic review and meta-analysis by Blanco-Colino et al. has shown that ICG fluorescence imaging seems to reduce AL rates following colorectal surgery for cancer [23].

### Post-operative care and follow up

Post-operative care and follow up will be as per institutional protocol, but patients must be reviewed at 30 days, and 6 months post-operatively at a minimum. Any further visits will be according to local standard clinical practice. All patients will be followed up as per protocol until 5 years after the last patient has been randomized.

### Statistical evaluation

#### Sample size

The primary endpoint is the pCR rate. Based on the published results from prospective studies on delayed time interval or observation only and on retrospective study for standard time interval, we assume that the mean rate of pCR in the standard treatment is about 15%, while the mean pCR rate in the observation treatment or longer time interval is 30%. To determine this difference, 270 patients are required, using a two-group continuity corrected χ^2^ test of equal proportions, assuming an α error of 4.9% and a power of 80% (MedCalc Version 17.9.7); an interim analysis on efficacy will be performed when half of events will be observed. The conservative Haybittle-Peto [24] boundary will be used as a stopping guidance in order to perform the final analysis at the significance level of 4.9%, two sides. Considering results from the pilot study reported on section 1, the percentage of unfavourable patients is 20% (favourable MRI tumor regression grade is defined as grades 1, 2 and 3; unfavourable MRI regression as grades 4 and 5). In addition, a meta-analysis on results from five randomized European clinical trials for locally advanced rectal cancer, has confirmed this rate of “poor” responders subgroup, identified by having no pCR and no DFS within 2 years [25]. In computing the sample size, we assume that the percentage of missing data will be 5%. A total of 340 patients, 170 for each arm, is intended to be enrolled, eventually. Patients will be randomized on a 1:1 basis to receive minimally-invasive rectal cancer surgery 8 or 12 weeks after neoadjuvant treatment and will be allocated a unique trial number. A computer-generated software with block randomization criteria will be used to ensure treatment groups are well-balanced for timing of surgery. All enrolled patients’ data will be registered in a prospective electronic database (ACCESS, MICROSOFT OFFICE Professional Plus 2010, regular licensed).

All data will be entered by means of case report forms. Original study forms will be entered and kept on file at the Coordinator site (SS. Antonio e Biagio e Cesare Arrigo Hospital). When a form is selected, the participating site staff will pull that form, copy it, and sent the copy to the DCC (Data Coordinating Center) for re-entry. Participant files are to be stored in numerical order and stored in a secure and accessible place and manner. Participant files will be maintained in storage for a period of 5 years after completion of the study.

The DCC will send monthly email reports with information on missing data, missing forms, and missing visits. Personnel at the Core Coordinating Center and the Participating Sites should review these reports for accuracy and report any discrepancies to the DCC.

### Statistical analysis

All efficacy outcomes will be assessed in the intention-to-treat population, which includes all enrolled patients who did not violate the eligibility criteria. pCR, OS and DFS will be assessed from the time of treatment allocation to local progression, death or disease progression. Patients who will not die and will not experience local of distant disease progression at the date of study cutoff will be censored at the last available information on status.

Time-to-event data will be analyzed by the Kaplan-Meier method and compared with the log-rank test. Cox proportional hazards model will be used to adjust the treatment effect for baseline prognostic factors.

### Serious adverse events reporting (SAE)

Any SAE considered to be reasonably related to the investigational treatment or study participation, have to be promptly notified.

This must be done by email within 24 h of the initial observation of the event. The principal investigator will decide if these events are related to the trial treatment (i.e. unrelated, likely related, and not assessable) and the decision will be recorded on the Serious Adverse Event form, if necessary with the reasoning of the principal investigator.

The investigator is obligated to assess the relationship between investigational treatment and the occurrence of each AE/SAE. A “reasonable possibility” is meant to convey that there are facts/evidence or arguments to suggest a causal relationship, rather than a relationship cannot be ruled out. The investigator will use clinical judgement to determine the relationship. Alternative causes, such as natural history of the underlying diseases, concomitant therapy, other risk factors, and the temporal relationship of the event to the investigational product will be considered and investigated.

### End of the study

The end of the study is defined as 5 years after the date that the last patient has been randomized to the trial.

## Research ethics approval

The protocol, site-specific informed consent forms, participant education and recruitment materials, and other requested documents — and any subsequent modifications — also has been reviewed and approved by SS. Antonio e Biagio e Cesare Arrigo Hospital Ethical Committee on 31 May 2018.

## Discussion

To date, it is well-know that pCR is associated with excellent prognosis and an overall survival of 90% [1]. In the Lyon trial the rate of pCR or near pCR increased from 10.3 to 26% [2] and in retrospective studies the increase rate was about 23–30%. These results may be explained on the relationship between radiation therapy and tumor regression: DNA damage occurs during irradiation, but cellular lysis occurs within the next weeks [3]. In the Stockholm III trial, a significantly lower frequency of postoperative complications was reported, even though not described in the other studies where morbidity and complications were the same.

There are several audiences for this trial: Oncologists, Surgeons, Radiation oncologists, Patients and the public, Academia, General Practitioners.

Another crucial point of the trial is the use of a structured MRI report, as recommended by the European Society of Gastrointestinal and Abdominal Radiology (ESGAR) [22], for primary staging and for restaging after neoadjuvant treatment. One of the goals of the trial is to determine whether MRI can specifically depict cancer local diffusion and predict downstaging and be used as a good prognostic instrument. High quality MRI, indeed, allows further subclassification of cT3, which is recommended by European Society for Medical Oncology (ESMO) guidelines and it is useful in stratifying and selecting patients with indication to neoadjuvant treatment before surgery.

In summary, the optimal interval between adjuvant chemoradiation and surgery may give the opportunity to optimize patients, initiate an individualized and “targeted” treatment, and favor organ preservation.

TiMiSNAR (NCT3465982 – https://www.timisnar.it) results, whether confirmed that performing surgery after 12 weeks from neoadjuvant treatment is advantageous from a technical and oncological point of view, may change the current pathway of the treatment in those patient suffering from rectal cancer.

## Data Availability

NOT APPLICABLE (the current manuscript doesn’t contain any data related to patients; it’s only a draft).

## Abbreviations

CT: Computed Tomography
DFS: Disease-free survival
DNA: DeoxyriboNucleic Acid
ESGAR: European Society of Gastrointestinal and Abdominal Radiology
ICG: Indocyanine Green test
IMRT: Intensity Modulated Radiotherapy
MRI: Magnetic Resonance Imaging
NOS: The Newcastle-Ottawa Scale
OS: Overall Survival
pCR: pathologic Complete Response
QoL: Quality of Life
RCT: radiochemotherapy
TaTME: transanal total mesorectal excision
TME: Total Mesorectal Excision
ymrTRG: MRI-based tumor regression grading
ypTRG: pathologic tumor regression grading
ESMO: European Society for Medical Oncology
