# Correction to: Standard (8 weeks) vs long (12 weeks) timing to minimally-invasive surgery after NeoAdjuvant Chemoradiotherapy for rectal cancer: a multicenter randomized controlled parallel group trial (TiMiSNAR)

**DOI:** 10.1186/s12885-020-6632-y

**Published:** 2020-02-17

**Authors:** Igor Monsellato, Filippo Alongi, Elisa Bertocchi, Stefania Gori, Giacomo Ruffo, Elisa Cassinotti, Ludovica Baldari, Luigi Boni, Graziano Pernazza, Fabio Pulighe, Carlo De Nisco, Roberto Perinotti, Emilio Morpurgo, Tania Contardo, Enzo Mammano, Ugo Elmore, Roberto Delpini, Riccardo Rosati, Federico Perna, Andrea Coratti, Benedetta Menegatti, Sergio Gentilli, Paolo Baroffio, Piero Buccianti, Riccardo Balestri, Cristina Ceccarelli, Valter Torri, Davide Cavaliere, Leonardo Solaini, Giorgio Ercolani, Elena Traverso, Vittorio Fusco, Maura Rossi, Fabio Priora, G. Numico, Paola Franzone, Sara Orecchia

**Affiliations:** 1 Azienda Ospedaliera SS. Antonio e Biagio e Cesare Arrigo, Alessandria, Italy; 2 Ospedale Sacro Cuore Don Calabria, Negrar, Italy; 3 Department of Surgery, Fondazione IRCCS Ca’ Granda, Ospedale Maggiore Policlinico, University of Milan, Milan, Italy; 4 Azienda Ospedaliera San Giovanni Addolorata, Rome, Italy; 5 Ospedale San Francesco, Nuoro, Italy; 6 Ospedale degli Infermi, Biella, Italy; 7 Ospedale Civile Pietro Cosma, Camposampiero/Ospedale Sant’Antonio, Padova, Italy; 8 Ospedale Civile Pietro Cosma, Padova, Camposampiero Italy; 9 Ospedale San raffaele IRCCS, Milan, Italy; 10 Azienda Ospedaliero Universitaria Careggi, Florence, Italy; 11 Azienda Ospedaliero Universitaria Maggiore Della Carità, Novara, Italy; 12 Azienda Ospedaliero Universitaria Pisana, Pisa, Italy; 13 Istituto di Ricerche Farmacologiche Mario Negri IRCCS, Milan, Italy; 14 Ospedale G.B. Morgagni L. Pierantoni, Forlì, Italy

**Correction to: BMC Cancer**

**https://doi.org/10.1186/s12885-019-6271-3**

Following publication of the original article [1], the authors reported that the family name of the author, Ludovica Baldari, was misspelled.

*Incorrect:*

Ludovica Baldarti

*Correct:*

Ludovica Baldari
